# Innovation ambidexterity and knowledge redundancy: The moderating effects of transactional leadership

**DOI:** 10.3389/fpsyg.2022.1003601

**Published:** 2022-09-29

**Authors:** Yunlong Duan, Wenjing Liu, Shanshan Wang, Meng Yang, Chang Mu

**Affiliations:** ^1^Division of Science & Technology Administration, Yunnan University of Finance and Economics, Kunming, China; ^2^International Business School, Yunnan University of Finance and Economics, Kunming, China; ^3^College of Innovative Business and Accountancy, Dhurakij Pundit University, Bangkok, Thailand; ^4^Business School, Yunnan University of Finance and Economics, Kunming, China; ^5^Business School, Central South University, Changsha, China

**Keywords:** knowledge redundancy, exploratory innovation, exploitative innovation, transactional leadership, knowledge management

## Abstract

Entering the challenging and promising knowledge era, it is clear that enterprises should leverage knowledge management activities in improving innovation performance to maintain competitive advantages. This study sheds light on the improvement path of innovation ambidexterity (i.e., exploratory and exploitative innovation) from the perspectives of knowledge redundancy and typical leadership style. It is noted that we determined the research theme through quantitative analysis and conducted qualitative analysis through 209 questionnaire data collected from respondents in different regions and industries in China. The empirical results indicated that knowledge redundancy significantly improves exploratory and exploitative innovation, and transactional leadership negatively moderates the above relationships. This study is of managerial implications to encourage employees to fully master and apply the existing knowledge to strengthen their innovation abilities in value creation. We also contribute to the theories pertaining to knowledge management, innovation, and ambidexterity by providing a deeper understanding of the influencing mechanism of knowledge redundancy in innovation ambidexterity while elaborating on the indirect effects of transactional leadership.

## Introduction

In the knowledge economy era, enterprises carry out knowledge management activities such as knowledge search, knowledge sharing, knowledge transfer, etc., to acquire new knowledge (Duan et al., 2020a), improve the economic benefits of enterprises (Haider and Kayani, 2020), achieve continuous innovation and enhance their core competitiveness (Jin et al., 2016). Nevertheless, it is obvious that these activities unavoidably accumulate a large number of redundant and repetitive knowledge, resulting in the generation of knowledge redundancy, in other words, the knowledge that an enterprise utilizes for its innovation processes is overlapping (Sivakumar and Roy, 2004). Furthermore, Moorman (2001) pinpointed that most enterprises would ignore the critical role of knowledge redundancy in the processes of knowledge sharing and knowledge transfer. In this vein, enterprises’ knowledge resources will be considerably wasted when the redundant knowledge fail to be effectively managed. Adversely, once the enterprise can better cope with the knowledge redundancy and avoid knowledge waste, their innovation performance is more likely to be promoted. Although existing studies have gradually attached attention to the role of knowledge redundancy in organizational performance (Stanko and Bonner, 2013), few scholars investigated the relationship between knowledge redundancy and innovation based on an ambidexterity perspective.

However, in today’s competitive and dynamic market environment, the production process of enterprises has become more and more complex and unstable. In this sense, enterprises are required to implement innovation ambidexterity to maintain long-term competitive advantages. Innovation ambidexterity refers to enterprises pursuing both exploratory and exploitative innovation simultaneously (He and Wong, 2004; Yang et al., 2015). Exploratory innovation refers to developing new products or new services based on current knowledge or new knowledge, while exploitative innovation refers to improving existing products or services on the basis of existing knowledge. However, enterprises that obsessively conduct exploratory innovation are likely to trap in endless search and unrewarding trials (Volberda and Lewin, 2003) and increase wasted resources. Likewise, an excessive pursuit of exploitative innovation may make the enterprise fall into the capability trap, causing difficulties in the adaptation to environmental changes. Compared with enterprises that only pursue either exploratory innovation or exploitative innovation, those that emphasize innovation ambidexterity are able to adapt to the environmental changes constantly, gaining high short-term performance while maintaining competitive edges in a long run. However, the simultaneous development of exploratory and exploitative innovation will inevitably result in competition for the resources of enterprises (Tushman and O Reilly, 1996; Raisch and Birkinshaw, 2008), which leads to the problem of resource shortage for enterprises. Therefore, when enterprises make strategic decisions on innovation activities, they need to strike a balance and allocate reasonable resource for both exploratory and exploitative innovation (He and Wong, 2004). As a part of enterprise resources (Leiponen, 2009), this study believes that knowledge redundancy is significant for enterprises to alleviate the suffering of limited knowledge, resolve the resource conflict between exploratory and exploitative innovation, and therefore improve the overall innovation ambidexterity. Therefore, we proposed that it is necessary to explore the influencing mechanism of knowledge redundancy on enterprises’ innovation ambidexterity, so as to provide significant insights into how to deal with the conflicts in ambidextrous pursuit of exploratory and exploitative innovation practice, which is conducive for managers and practitioners to leverage the redundant knowledge resources to create value through innovation.

Moreover, it is reported that leadership plays a crucial role in the development of enterprises. Different leadership styles have different impacts on knowledge management and innovation decision-making in enterprises (Trung et al., 2014; Zhang et al., 2015), and further affect the effect and efficiency of organizational activities (Alrowwad et al., 2020). Similarly, extant studies provided substantial evidence on that varied leadership styles will influence knowledge management activities such as knowledge sharing (Srivastava et al., 2006; Haider et al., 2021), knowledge transfer (Goh, 1994), knowledge hiding (Kim, 2021) and knowledge integration (Ghazali et al., 2015). In addition, scholars have reached a consensus that different leadership styles play a significant role in organizational innovation performance. Appropriate leadership styles create a good innovation atmosphere (Ekvall and Ryhammar, 2010), encourage subordinates to move toward the same goal (Zahoor et al., 2022), stimulate employees’ innovative vitality (Vecchio et al., 2010), and promote enterprise innovation. However, negative leadership styles are likely to inhibit employees’ intrinsic incentives for innovation, thereby discouraging the enterprise’s innovation performance (Pieterse et al., 2010). Therefore, this study believes that leadership styles play a key role in the success of an organization. Extant literature on leadership styles mainly focused on transactional or transformational leadership. In particular, transactional leadership is a typical leadership style in China. Transactional leadership means that the enterprise’s managers set up organizational goals based on the role clarification and job requirements, thereby leveraging differential rewards and punishments to motivate subordinates to complete the proposed job goals (Bass, 1999; Mackenzie et al., 2001), The focus of transactional leadership is to maintain and ensure the efficient operation of the organization’s daily work (Haider et al., 2021), which will have a certain impact on enterprises’ innovation performance (Makri and Scandura, 2010; Vargas, 2015; Jia et al., 2018). At the same time, scholars have provided empirical support that leadership style is significantly associated with knowledge management in enterprises (Bryant, 2003; Connelly and Kelloway, 2003; Yang and Chen, 2007; Hai and Mohamed, 2011), such as transformational leadership promotes knowledge sharing (Liu and Li, 2018). To conclude, most existing research elucidated the influence of transactional leadership on innovation, which however ignores the role of transactional leadership in the relationship between knowledge management and enterprises’ innovation ambidexterity. Therefore, this study builds up a research model to investigate the moderating role of transactional leadership in the relationship between knowledge redundancy and innovation ambidexterity.

In summary, there is a lack of in-depth studies that comprehensively explain the relationships among knowledge redundancy, innovation ambidexterity, and transactional leadership. Hence, we take Chinese enterprises as the research object, associated knowledge redundancy with the ambidexterity theory to establish a theoretical model of knowledge redundancy and innovation ambidexterity, in which transactional leadership style is involved as a moderating variable. In this way, this study attempts to supplement the research gaps by addressing the following questions: (1) Does knowledge redundancy significantly impact enterprises’ innovation ambidexterity (i.e., exploratory and exploitative innovation), positively or negatively? (2) Is there a moderating role of transactional leadership in the above relationships? The research results will provide theoretical support and methodological guidance for enterprises to realize dual innovation and maintain long-term competitive advantage.

Subsequent parts of this study are organized as follows. The second section elaborates on the relevant theoretical background of the principal variables, and then proposes the corresponding hypotheses. The third section introduces the methodology, including questionnaire design, the descriptive statistics of research data, as well as the reliability and validity analyses. The fourth section conducts the empirical analysis to examine the proposed hypotheses. Finally, the fifth section is the conclusion and discussion, in which we shed light on research conclusions, theoretical contributions, management implications, research limitation, and future prospects.

## Theoretical background and hypotheses development

### Knowledge redundancy

As an essential part of organizational redundancy (Sivakumar and Roy, 2004), knowledge redundancy is regarded as a type of resource redundancy in enterprises, which is of great organizational value to cultivating competitive advantages (Phelps, 2010). Redundancy was originally considered as the difference between the resources required by small groups in the enterprise and the resources actually required. The term knowledge redundancy was firstly proposed by Bourgeois (1981), drawing from an organizational theory perspective, redundancy was defined as an excess of resources that can be used at will to mitigate the external environmental changes. Along with the research theoretical development of knowledge redundancy, relevant scholars have analyzed knowledge redundancy in varied research context and objects from different perspectives. Moorman (2001) pointed out that knowledge redundancy is the similarity degree of the information, knowledge and skills mastered by the relevant participants on the enterprise’s new product development. Sivakumar and Roy (2004) contended that knowledge redundancy is a type of knowledge accumulation that usually exceeds the current needs for enterprises’ innovation, so it cannot be easily applied and is more likely to be neglected. Stanko and Bonner (2013) proposed that knowledge redundancy is the degree of knowledge overlap between customers and suppliers, while Expósito-Langa and Molina-Morales (2009) defined knowledge redundancy as the degree of overlap of knowledge bases between two or more social actors. To sum up, drawing from the viewpoints of Moorman (2001), Sivakumar and Roy (2004), etc., this study further defines knowledge redundancy as the overlap and similarity degree of the knowledge acquired by an enterprise’s employees during social activities and is currently beyond the required innovation resources.

It is evident that relevant scholars have investigated different organizational consequences of knowledge redundancy. However, the research findings were controversial considering the nature of redundancy (Jenssen and Greve, 2002). On the one hand, some scholars concluded that redundant resources are a waste of organizational resources, which will increase the management cost, hinder the knowledge flow within the organization, and reduce the effectiveness of innovation activities (Cheng and Kesner, 1997; Expósito-Langa and Molina-Morales, 2009). Moreover, enterprises with a high degree of knowledge redundancy will obtain less novel and diverse information (Moon et al., 2022). At this time, knowledge redundancy may have a negative impact on the breakthrough innovation of enterprises. On the other hand, some researchers hold that knowledge redundancy can promote the enterprises’ knowledge transfer, enhance knowledge absorption capacity, and improve knowledge utilization, thereby promoting the overall innovation performance (Gupta and Govindarajan, 2000; Moorman, 2001; Zahra and George, 2002). In addition, it is proposed that knowledge redundancy can be treated as a reserve resource that helps enterprises cope with emergencies (Gulati, 1996; Carnes et al., 2019) to adapt to environmental change successfully (Levinthal and March, 1993; Greenley and Oktemgil, 1998) Based on an extensive literature review, we concluded that knowledge redundancy has both positive and negative influences on organizational outcomes. Specifically, the positive viewpoints focus on its effects of enhancing the comprehension of organizational knowledge to realize the full extent of application, whereas those suggesting knowledge redundancy is detrimental highlight it will increase the management cost and inhibit the knowledge flow. But regardless of the result, the view that knowledge redundancy is an enterprise resource is generally accepted (Gulati, 1996; Herold et al., 2006).

### Innovation ambidexterity

Ambidexterity refers to an enterprise’s dual capabilities, that is, the enterprise can not only integrate and utilize the existing resources to improve the efficiency of goals and tasks but also expand external resources to purposefully gain competitive edge in a dynamic and challenging environment (Duncan, 1976). Based on the perspective of organizational learning, March (1991) proposed two different organizational learning behaviors called exploration and exploitation, respectively. After that, exploration and exploitation are regarded as a vital topic in the fields of organizational learning (March, 1991; Auh and Menguc, 2005), organizational adaptation (Burgelman, 1991, 2002), and technological innovation (Benner and Tushman, 2003; Atuahene-Gima, 2005). Besides, March (1991) defines exploration as organizational behaviors that experiment new choice, which usually result in uncertain, immediate, even negative results. He and Wong (2004) highlighted that exploration behavior is likely to produce new products or services through research, discovery, experimentation, divergent thinking, risk-taking, etc. On the other hand, March (1991) defined exploitation as to refine and expand the existing capabilities, technologies and paradigms, which results in slight, gradual, and incremental improvement of existing products and services. Benner and Tushman (2003) applied these two types of behaviors to innovation research, in which enterprises’ innovation is divided into two categories, namely exploratory and exploitative innovation. Exploratory innovation is the process of creating new goods and services by acquiring new knowledge or further integrating existing knowledge. Exploitative innovation refers to the enhancement of current goods and services based on the enterprises’ existing knowledge and information to fulfill the needs of the enterprises’ current customers (Lin and Chang, 2015). Furthermore, we found that relevant researches also divided innovation into incremental and breakthrough innovation (Christine et al., 2003). Incremental and breakthrough innovation mainly emphasize on the *ex post* evaluation regarding the degree of innovation achieved by technological achievements, whereas exploratory and exploitative innovation focus on the *ex ante* learning behavior within an enterprise, which reflects the enterprise’ attitude toward explore—exploit innovative activities. To conclude, in accordance with Brion (2010), this study considers innovation ambidexterity as two types of activities, namely exploratory and exploitative innovation, to highlight their differential outcomes. It is noted that the literature of exploratory and exploitative innovation is still in the exploratory stage, while there is limited knowledge concerning how to strike a balance between the two-dimensional innovative activities. Furthermore, prior studies that associate knowledge management with exploratory and exploitative innovation has mainly focused on the knowledge heterogeneity (Tsai, 2016), knowledge sharing (Yi et al., 2019; Berraies et al., 2020; Zhang et al., 2022), and knowledge transfer (Yin et al., 2019), yet little is known about how knowledge redundancy interacts with exploratory and exploitative in enterprises. In light of this, we shed light on the effects of knowledge redundancy on exploratory and exploitative innovation, respectively, aiming at extends existing research regarding knowledge management and innovation ambidexterity.

### Knowledge redundancy and innovation ambidexterity

Katila and Shane (2005) pointed out that the knowledge acquired by enterprises cannot be smoothly transformed into innovation performance. In other words, as knowledge is acquired and applied by enterprises, certain knowledge redundancy will be generated, which will be converted into organizational resources. Then, the enterprises need to consume a certain number of resources to carry out both exploratory and exploitative innovation. It is noted that knowledge redundancy is a reserve resource, which can alleviate the resource competition due to the implementation of innovation ambidexterity in case of increasing resource constraints. Therefore, we contend that knowledge redundancy is likely to promote enterprises’ innovation ambidexterity and the major reasons are as follows.

### Knowledge redundancy and exploratory innovation

First, knowledge redundancy can be regarded as an essential organizational resource to promote exploratory innovation. On the one hand, it is clear that enterprises’ resources play a crucial role in the processes of new product development. As a kind of organizational resource, knowledge redundancy is able to provide enterprises with the knowledge resources needed to implement exploratory innovation (Penrose, 1996; Leiponen, 2009), which enables them to have sufficient resources and abilities to experiment new project ideas, develop new products, thereby promoting exploratory innovation performance. On the other hand, scholars have provided evidence that suggest enterprises with a higher level of knowledge redundancy have more idle resources, and at this time enterprises have stronger environmental adaptability (Levinthal and March, 1993) and risk tolerance (Gulati, 1996; Moreno et al., 2009), especially in terms of a resource-constrained environment, knowledge redundancy enables enterprises to acquire new knowledge through internal knowledge mining, which is conducive to reducing the cost of exploratory innovation, strengthening the ability to resist risks, so as to promote the exploration and development of high-risk projects. Moreover, it is noted that the idle resources can be used as buffer resources to moderate the conflicts among internal goals (Cyert and March, 1963), promote the rational allocation of organizational resources, and alleviate the resource constrains of explore–exploit activities, thereby promoting exploratory innovation.

Second, knowledge redundancy is significant to expanding enterprises’ knowledge depth and scope. Hagedoorn and Cloodt (2003) pinpointed the generation of knowledge redundancy indicating there are more diverse types of knowledge in the enterprises, which enriches the existing knowledge base. In this sense, diverse knowledge brings additional new ideas and thoughts to enterprises, which is more likely to develop new methods and skills to solve a certain problem (Ahuja and Morris Lampert, 2001). Furthermore, a high level of knowledge redundancy is a sign that the enterprises possess more heterogeneous knowledge, and heterogeneous knowledge is a key factor for enterprises to engage in exploratory innovation to maintain core competencies (Harvey, 2013). That is to say, heterogeneous knowledge help enterprises generate creative thinking to promote exploratory innovation (Suzuki and Kodama, 2004).

Third, knowledge redundancy positively facilitates the reconfiguration of professional workforce. When knowledge redundancy arises, enterprises will take the initiative to reintegrate the current workforce or set up a rotation system to establish a good talent flow (Scott, 2012), which prompts employees to unshackle themselves from rigid thinking and increase their intrinsic motivation to innovation. Additionally, we hold that the essence of innovation is to generate new ideas and opportunities through the progressive accumulation and recombination of existing and new knowledge (Ali et al., 2021), in the process of job rotation, innovative employees can acquire varied and complex knowledge, enhance their technological innovation ability and promote exploratory innovation.

Based on the above analysis, this study proposes the following hypothesis:

*H1: Knowledge redundancy positively affects the exploratory innovation in enterprises.*

### Knowledge redundancy and exploitative innovation

First, knowledge redundancy could provide enterprises necessary resources to improve their product and innovation process. Unlike exploratory innovation that creates new product and services, exploitative innovation focuses on making improvement of the enterprises’ existing products or services based on their customers’ needs. As a result, exploitative innovation is less complicated and relies more on existing information and knowledge. A high level of knowledge redundancy indicates that the enterprise has untapped knowledge resources, suggesting a greater knowledge stock. Prior research studies made a consensus that knowledge redundancy could provide new knowledge elements for exploitative innovation (Leiponen, 2009), because the mining of existing and unused knowledge resources for knowledge re-creation not only reduce the cost regarding knowledge search and knowledge transfer but also enables the enterprises to make full use of existing knowledge base while maximizing the value of idle resources. Furthermore, Colberg et al. (1964) underlined that knowledge redundancy alleviates the resource constraints in carrying out innovation ambidexterity, as it can provide required knowledge and information for exploitative activities, thereby promoting enterprises’ exploitative innovation.

Second, knowledge redundancy enriches the enterprises’ original knowledge base. To be more specific, knowledge redundancy strengthens an enterprise’ heterogeneous knowledge reserve by increasing the quantity and variety of organizational knowledge. Therefore, heterogeneous knowledge provides more creativity for enterprises to improve existing products and services. The higher degree of knowledge redundancy an enterprise has, the more types of knowledge the enterprise will master, which indicates there will be a higher level of knowledge heterogeneity. In this case, heterogeneous knowledge enriches the knowledge elements owned by enterprises and enhances the knowledge creation level of enterprises (Liu et al., 2018; Gao et al., 2019; Hou et al., 2021). Meanwhile, knowledge redundancy also increases the opportunities for the enterprises to integrate and recombine varied knowledge, thereby producing a greater number of available approaches and strategic decisions to for exploitative innovation (Ahuja and Morris Lampert, 2001).

Third, knowledge redundancy improves enterprises’ ability in knowledge application to create more resource for exploitative innovation. When there is knowledge redundancy, the enterprise would have a deep understanding of their knowledge base, so they are more likely to allocate the most valuable knowledge to specific innovative activities. Additionally, enterprises with a high level of knowledge redundancy usually have established a strong ability to make full use of available knowledge resources. In this sense, as knowledge application is significant for enterprises to achieve an incremental upgrading regarding the existing products or services (Duan et al., 2020b), it is apt to conclude that knowledge redundancy promotes the knowledge application of enterprises, and then promotes the exploitative innovation. Specifically, enterprises with enhanced ability in knowledge application have advantages in integrating both internal and external resources (Teece et al., 2009), which empowers them to fully tap the potential of knowledge, broaden the scope of knowledge base, improve resources utilization efficiency, thereby create additional value through exploitative innovation (Majchrzak and Neece, 2004).

Based on the above analysis, this study proposes the following hypothesis:

*H2: Knowledge redundancy positively affects exploitative innovation in enterprises.*

### The moderating effect of transactional leadership

Burns first proposed the transactional leadership style. Burns pointed out that transactional leaders pay attention to the exchange relationship between themselves and their subordinates. Leaders and subordinates meet their own needs through interest exchange and achieve mutual benefit through negotiation. At the same time, transactional leaders follow the principle of maximizing their own interests and minimizing losses (Burns et al., 1978). Burns et al. (1978) proposed that the core of transactional leadership lies in a leader-member relationship exchange relationship of “bargaining for things” and “clear rewards and punishments.” Meanwhile, Bass (1985) developed a theory concerning transactional leadership, suggesting that transactional leadership is adapted from the Leadership-Member Exchange Theory (LMX) and Path-Goal Theory. Bass defines transactional leadership as a kind of transaction process that meets the needs of both parties through “clarifying the role orientation—clarifying the work requirements—supervising the work process—giving rewards to subordinates according to the results” (Bass, 1985). Hollander (1978) underlined that transactional leadership relies on the principle of exchange and the subordinates are rewarded through accomplishing specific goals (Bass and Avolio, 1990), hence the subordinates would neither exceed the initial expectations nor be motivated to try creative ideas or solutions that may alter the *status quo* (Jung, 2001). In other words, it is likely that transactional leadership is not conducive to corporate creativity (Amabile, 1998; Yi et al., 2019). As studied by Vigoda-Gadot (2007) and Rank et al. (2009), transactional leadership negatively associated with employees’ innovation performance, because this leadership style usually plays a role in a simple, stable, and predictable working environment. Based on this, this study further investigated the indirect effects of transactional leadership on the relationships between knowledge redundancy and innovation ambidexterity.

### The moderating effect of transactional leadership on knowledge redundancy and exploratory innovation

This study proposed that transactional leadership plays a negative moderating role in the relationship between knowledge redundancy and exploratory innovation for the following reasons.

First, transactional leadership makes enterprises to be too risk averse to innovate radically. Transactional leaders would establish clear goals for their subordinates, closely and actively monitor their actions (Bass et al., 2003) while asking them to perform their work in accordance with the enterprise’s rules and regulations. When the subordinates make work mistakes, they will be severely punished and the consequences will be reflected in their compensation (Pillai et al., 1999). Given that transactional leaders are risk-averse leaders and will inhibit unpredictable risky projects (Jansen et al., 2009). In this way, transactional leadership encourages subordinates to follow the leader step by step instructions and earn the corresponding compensation rewards to avoid making mistakes at work and receiving negative consequences, as a result they are less likely to proactively try innovative proposals with high risks (Berraies and Bchini, 2019). However, since exploratory innovation is characterized by taking high risks and making fundamental changes (March, 1991), it is clear that transactional leadership inhibits employees from generating and exchanging disruptive ideas (Mccleskey, 2014) for exploratory innovation.

Second, transactional leadership hinders the interaction and information exchange among employees. Transactional leadership emphasizes the exchange relationship between the leaders and subordinates (Sergiovanni, 1990) and the leader will give certain rewards to subordinates according to their task performance. Hence, it is concluded that transactional leadership belongs to a task-oriented leadership, under which the subordinates will take various measures to complete the tasks assigned by the leader on time in exchange for their leaders’ incentive. At this time, the subordinates would concentrate on completing the tasks, thus they work in a low-autonomy work environment. Additionlly, according to LMX, task-oriented leadership style affects the trust level of leaders and subordinates (Spender, 1996), which discourages the knowledge exchange and transfer within and outside the enterprises. Nevertheless, leader and managers need to give their subordinates greater individual autonomy for innovative activities, so they are able to exchange and share their own heterogeneous knowledge. Therefore, subordinates are likely to engage in less knowledge exchange under transactional leadership style, and when knowledge redundancy is at a lower level, enterprises would lack new knowledge and technology and face more severe knowledge resource constraints for exploratory innovation (Phene et al., 2012).

Based on the above analysis, this study proposes the following hypothesis:

*H3: Transactional leadership negatively moderates the relationship between knowledge redundancy and exploratory innovation.*

### The moderating effect of transactional leadership on knowledge redundancy and exploitative innovation

This study posited that transactional leadership plays a negative moderating role in the relationship between knowledge redundancy and exploitative innovation for the following reasons.

First, transactional leadership reduces the employees’ willingness to plan and implement exploitative innovation. Transactional leadership is so benefit-oriented, it follows the principles of exchange and maximizing interests (Burns et al., 1978) and will promote a task-compensation exchange relationship between leaders and subordinates to make sure that the employee rewards depend on the amount of contribution. Different from exploratory innovation, exploitative innovation emphasizes mining and integrating existing knowledge to optimize the enterprise’s existing products or services (Sariol and Abebe, 2017), may have lower risks and returns. In this sense, employees are less likely to acquire, integrate and advance the existing knowledge to implement exploitative innovation, because they cannot meet the expected salary by implementing exploitative activities with low returns. Therefore, transactional leadership causes subordinates to lack intrinsic motivation for exploitative activities that are subjective and self-motivated (Chen et al., 2010), and they would rather invest their knowledge and resources to other organizational activities with higher returns.

Second, transactional leadership style hinders positive knowledge sharing and exchange within an enterprise. Considering it especially pays attention to organizational goal achievement, transactional leadership always promote transactional psychological contracts between leaders and subordinates, as well as subordinates and subordinates (Politis, 2001), which negatively influences the degree of trust and effective communication among innovation contributors (Brower et al., 2000). Relatedly, it is argued that transactional relationships discourage a favorable atmosphere for innovation and reduces the willingness of knowledge sharing and exchange among employees (Bouty, 2000). At the same time, since knowledge redundancy frequently results from knowledge exchange inside and outside the enterprise, a lower level of knowledge sharing caused by the transactional leadership style reduces the likelihood of knowledge redundancy, the reserve of heterogeneous knowledge resources, and the knowledge application for exploitative innovation.

Based on the above analysis, this study proposes the following hypothesis:

*H4: Transactional leadership negatively moderates the relationship between knowledge redundancy and exploitative innovation.*

**FIGURE 1:**
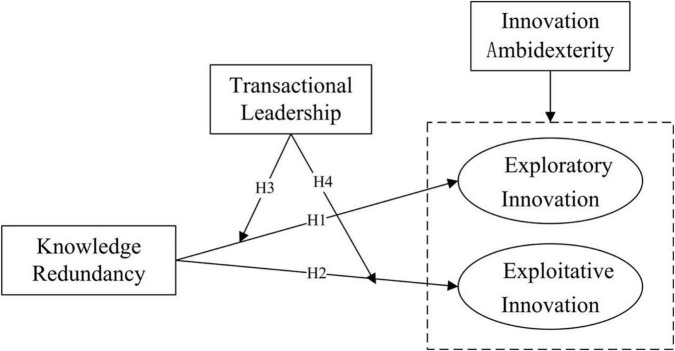
Conceptual model.

To conclude, our research model is shown in Figure 1.

## Materials and methods

### Sample and data

In order to ensure the accuracy of the research content, we managed to conduct in-depth qualitative interviews with the front-line professional employees and managers before the formal survey to identify this study’s research topic. Specifically, we interviewed the following questions: (a) how to view knowledge redundancy? (b) Do you think there is knowledge redundancy in the company? (c) Does the leader of the company often communicate with his subordinates? Then, we determined the principal variables, integrated the relevant questionnaire items, arranged the procedure for data collection to implement the following quantitative analysis. More specifically, this study utilized an advanced questionnaire to collect research data from employees in different industries across varied regions (i.e., the eastern, central, and western areas) in China to ensure the diversity of research subjects. Manufacturing industry is the main object of this survey. This questionnaire is mainly designed as an electronic questionnaire, which is distributed through e-mail. The questionnaire survey lasted for three months from December 2021 to February 2022. A total of 335 questionnaires were distributed. After excluding questionnaires that were incomplete and inconsistent or were completed in a short time, 209 valid questionnaires were obtained for empirical analysis with a response rate of 62.39%. The descriptive statistics of the research sample is displayed in Appendix Table A1. In detail, 47.4% were female and 52.6% were male. In terms of the job position, 41.4% of the respondents were general employees, while lower-level, middle-level and senior managers accounts for 23.9, 23.9, and 10%, respectively. For job tenure, despite 13.9% of respondents have worked for less than one year, the other respondents have accumulated more extended working experience. In addition, the proportion of the enterprise size in the research sample is 18.2% (<20 employees), 30.6% (20–100 employees), 18.2% (100–300 employees) employees, and 33% (>300 employees), respectively. As for the ownership, there are 49.8% of respondents worked in state-owned firms, and those who worked in collective or private firms account for 31.6%. Overall, it is clear that the research sample with a reasonable structure covered a relatively wide range, meeting the research design requirements.

### Measures

All questionnaire items of the variables, unless stated otherwise, were measured on a Likert 5-point scale that ranges from 1 = strongly disagree to 5 = strongly agree. First, based on an extensive literature review, we collected and analyzed questionaries that had been validated in previous relevant literature. Second, considering the original items of principal variables were designed in English, back-translation procedure was applied to translate the original questionnaire items into Chinese. Finally, all items were tailored and simplified so that respondents in specific Chinese contexts could easily understand and evaluate them.

### Dependent variable

The dependent variables are exploratory and exploitative innovation (**EI, LI**). The measurement instruments of exploratory and exploitative innovation consist of three items, respectively, which were adapted from Jansen et al. (2006) and Hughes et al. (2010). The sample items for exploratory innovation include “We always develop new products and services,” and “We often experiment with new products and services in the market,” presenting the values of factor loading ranging from 0.840 to 0.855. Meanwhile, the sample items for exploitative innovation include “We regularly introduce improved products and services to the market,” and “We regularly make small adjustments to existing products and services.” The values of the factor loadings ranged from 0.732 to 0.876.

### Independent variable

The independent variable is knowledge redundancy (**KR**). The measurement instrument is adapted from Expósito-Langa and Molina-Morales (2009), Morales and Langa (2013), and Stanko and Bonner (2013). The sample items include “The external and internal knowledge exchange in your enterprise have a certain similarity,” and “Your enterprise will conduct more knowledge exchange activities at the individual and firm-level than other cooperative enterprises.” The values of the factor loadings ranged from 0.682 to 0.839.

### Moderating variable

The moderating variable is transactional leadership (**TL**). The measurement instrument of transactional leadership with five items were referred to previous studies of Waldman et al. (2001) and Alrowwad et al. (2020). The sample items include “My leader will severely punish me because of work faults and negative behavior,” and “My enterprise’s leadership style will require employees to act by organizational rules.” The values of the factor loadings ranged from 0.612 to 0.836.

### Control variables

Gender **(Gen)**. We controlled for gender because it is acknowledged that females and males present specific differences in thinking mode and innovation awareness, they would present their own advantages and disadvantages in different aspects of innovation implementation (He and Wong, 2011). In this regard, employee gender is likely to impact on the performance of exploratory and exploitative innovation, so we select gender as a control variable.

Job Tenure **(JT)**. The longer an individual employee’ s tenure with an enterprise, the more likely they would resist change (Majchrzak and Neece, 2004), thus hindering them to conduct exploratory innovation. Therefore, we controlled for the job tenure in this study. Specifically, job tenure was measured by the following scales: <1 year, 1–3 years, 4–6 years, 5–9 years, and 10+ years.

Job Position **(JP)**. Employees in different job positions have varied organizational autonomy, so they will perform differently in knowledge acquisition and innovative activities, and their impact on innovation is also different (Duan et al., 2021). As a result, job position was selected as a control variable and was measured based on four categories: general staff, low-level managers, middle-level managers, and senior managers.

Enterprise Scale **(ES)**. It is clear that enterprise size significantly affects the level of knowledge resources utilization and the development of innovation strategies. Larger enterprises have both the organizational resources and excellent risk management capabilities to engage in a variety of innovation-related activities (Chin et al., 2021). Duan et al. (2020a) pinpointed small scale enterprises would be more flexible in formulating innovation strategies. Following this, we concur that ES is closely associated with the outcomes of exploratory and exploitative innovation. There are four categories in ES measurement, namely less than 20, 20–100, 101–300, and more than 300 employees.

R&D Investment **(R&D)** There is evidence that R&D investment is a positive facilitator for innovation ambidexterity, because it provides essential supports for innovation activities in an enterprise (Scherer, 1965; Hall et al., 2013). Moreover, we highlight that R&D investment intensity reflects the enterprise’ attitude to innovation, since it is a critical fund source of innovation strategies. Therefore, R&D investment is selected as a control variable.

Ownership **(Owner)**. We controlled for organizational ownership because different organizational type potentially affects the quantity of available organizational resources, organizational atmosphere, and professional workforce for innovation (Duan et al., 2022). Likewise, different types of enterprises have differential motivations and necessity in innovation. In this way, we measured ownership in five categories: state-owned enterprises, private enterprises, Sino-foreign joint ventures, wholly foreign-owned enterprises, and other types of enterprises.

## Results

### Confirmatory factor analysis

We have performed Kaiser-Meyer-Olkin (KMO) test and Bartlett’s sphericity test on the sample data before the factor analyses. The results showed that the value of KMO was 0.885 (greater than the critical value 0.5), and all principal variables passed Bartlett’s sphericity test (*p* < 0.001). Furthermore, MPLUS 8.0 was applied to verify the research model’s convergent validity and the discriminant validity. In terms of the conduct confirmatory factor analysis (CFA), the entire model fitting results proved that several indicators without the CFA modification are unacceptable according to the numerical values of the indicators. After applying the Modification Indices (MI), it is found that the fitting degree of the research model was up to standard. Given that χ^2^ = 118.71, DF = 98, χ^2^/DF = 1.211 (1 < χ^2^/DF < 3), CFI = 0.989, TLI = 0.986 (>0.9), RMSEA = 0.032, SRMR = 0.045 (<0.08), the model presents a good convergent validity and the model fit data is shown in Table 1.

**TABLE 1:** Model fitting index.

Index	Criteria	Value	Fit
ML χ^2^	The smaller the better	118.71	
DF	The bigger the better	98	
χ^2/^DF	Between 1–3	1.211	Fit
CFI	Greater than 0.9	0.989	Fit
TLI	Greater than 0.9	0.986	Fit
RMSEA	Less than 0.08	0.032	Fit
SRMR	Less than 0.08	0.045	Fit

### Reliability and validity tests

The validity test aims at attesting the convergent validity and the discriminant validity. We measured the convergent validity based on three indicators, namely the values of Squared Multiple Correlation (SMC), Composite Reliability (CR), and Average Variance Extracted (AVE), which examines the consistency, stability and reliability of the data, respectively. First, SMC elucidates the dimensions regarding the questionnaire items, which is the square of the estimated value of the R-square. If SMC > 0.36, it is acceptable. If SMC > 0.5, it means that the SMC is good. Second, SMC in the Table 2 are all greater than the threshold value 0.36, indicating that the proposed dimension has a strong explanatory power to the research topic. Second, CR tests the internal consistency about the dimensions and questionnaire items. In this study, the values of CR are all greater than 0.867, suggesting there is aa high degree of internal consistency. Moreover, AVE refers to the degree of explanation concerning the dimensions of the items. In this study, the AVE values of each principal variables are between 0.582 and 0.723, all exceeding the recommended threshold value 0.36, thereby indicating that the research data has high convergent validity and reliability.

**TABLE 2:** Reliability and convergent validity table.

Var	Item	Parameters of significance test				Item reliability	Composite reliability	Convergence validity
		Estimate	SE	Est./SE	*P*-value	R-square	CR	AVE
KR	KR1	0.732	0.038	19.376	***	0.536	0.877	0.588
	KR2	0.812	0.030	26.948	***	0.659		
	KR3	0.839	0.028	30.312	***	0.704		
	KR4	0.759	0.035	21.466	***	0.576		
	KR5	0.682	0.042	16.136	***	0.465		
EI	EI1	0.840	0.028	29,929	***	0.706	0.887	0.723
	EI2	0.855	0.027	31.536	***	0.731		
	EI3	0.855	0.027	31.572	***	0.731		
LI	LI1	0.869	0.029	29.572	***	0.755	0.867	0.686
	LI2	0.876	0.029	30.137	***	0.767		
	LI3	0.732	0.038	19.291	***	0.536		
TL	TL1	0.691	0.041	16.781	***	0.477	0.873	0.582
	TL2	0.612	0.048	12.735	***	0.375		
	TL3	0.832	0.028	29.612	***	0.692		
	TL4	0.817	0.029	28.015	***	0.667		
	TL5	0.836	0.028	30.091	***	0.699		

***P < 0.001. KR, knowledge redundancy; EI, exploratory innovation; LI, exploitative innovation; TL, transactional Leadership.

In order to determine the discriminant validity among latent variables, it is necessary to compare the values of the square root of AVE with the correlation coefficients between latent variables, and the former should be greater than the latter. Table 3 concludes the results of reliability, convergent validity, and discriminant validity, and it is shown that this study’s four latent variables have met the criteria and prove the high validity of our research questionnaire.

**TABLE 3:** Convergent validity and discriminant validity.

Var	Std.Loading	Compote reliability	Conference validity	Discrimanate validity			
		CR	AVE	KR	EI	LI	TL
KR	0.682–0.839	0.877	0.588	**0.767**			
EI	0.840–0.855	0.887	0.723	0.419	**0.850**		
LI	0.732–0.876	0.867	0.686	0.216	0.823	**0.828**	
TL	0.612–0.836	0.873	0.582	0.223	0.235	0.295	**0.763**

The bold font is the square root of AVE, and the lower triangle is the Pearson correlation coefficient of the variable. Among them, KR, knowledge redundancy; EI, exploratory innovation; LI, exploitative innovation; TL, transactional leadership.

### Common method deviation test

For common method deviation, the following methods are adopted to test. First, adopt the Hamann one-way test. Put all latent variables into the factor analysis without rotation, and observe whether the principal component of the first precipitation is lower than 50% (Ali et al., 2021). The results showed that the first principal component explanation accounted for 34.48%, so the Hamann one-way test passed. Second, the correlation coefficient of each latent variable is used to determine whether there is a serious common method deviation problem. If the correlation coefficient of each potential variable exceeds 0.9, it is considered that the common method deviation problem is serious; If the correlation coefficient of each potential variable is lower than 0.9, it indicates that the common method deviation problem is acceptable. It can be seen from Table 3 that the correlation coefficient of each potential variable in this paper does not exceed this standard. Third, we examined the competing model fitting indices of the confirmatory factors to test the discriminant validity between the principal variables. It is shown in Table 4 that the 3-factor model, 2-factor model, and single-factor model all performed poorly, while the 4-factor model with strong discriminant validity is better than other alternative models when refers to the values of the fitting indicators (i.e., χ^2^ = 118.71, DF = 98, χ^2^/DF = 1.211, CFI = 0.989, TLI = 0.986, RMSEA = 0.032, and SRMR = 0.045). Therefore, this study has no significant issues about common method bias.

**TABLE 4:** Competitive model fit index.

Models	χ ^2^	DF	χ ^2/^DF	CFI	TLI	RMSEA	SRMR
(Benchmark model) 4–Factor model: KR, EI, LI, and TL	118.710	98	1.211	0.989	0.986	0.032	0.045
3-Factor model: KR + EI, LI, and TL	123.894	99	1.251	0.987	0.984	0.035	0.059
2-Factor model: KR + EI + LI, and TL	129.966	100	1.299	0.984	0.981	0.038	0.062
Single-factor model: KR + EI + LI + TL	129.966	100	1.300	0.984	0.981	0.038	0.062

*N* = 209.

### Hypothesis tests

According to the model and research hypotheses, regression analyses were performed on the main effect, and the moderating effect models, respectively. First, we regressed knowledge redundancy on exploratory and exploitative innovation to verify the proposed hypotheses about the main effects. Then, we examined the moderating effect of transactional leadership on the relationships between knowledge redundancy and exploratory and exploitative innovation, respectively.

### Main effects

As shown in Table 5, Model 1 and Model 2 contains only the control variables, namely the Gen, JT, JP, ES, Owner, and R&D. Model 3 and Model 4 tests the main effects: H1 and H2. Specifically, Model 3 verified the effect of knowledge redundancy on exploratory innovation of enterprises (H1). Given that regression coefficient of the model is 0.454, *p* < 0.001, indicating that EI was improved with the increase of KR. Therefore, H1 was verified. When KR increases by 1 unit, EI increases by 0.454 units, which means that when an enterprise has a high level of knowledge redundancy, it will have more organizational resources to try new ideas while the enterprises’ employees are strongly encouraged to carry out exploratory innovation. Meanwhile, Model 4 verified the impact of knowledge redundancy on exploitative innovation (H2). The regression coefficient of the model is 0.254, *p* < 0.01, indicating that LI was promoted with the increase of KR, suggesting H2 was verified. When KR increases by 1 unit, LI increases by 0.254 unit. It is explained that when the level of knowledge redundancy is high, there are utilized knowledge resources in the enterprise, so that it can deeply mine and integrate the existing knowledge, thereby improving exploitative innovation.

**TABLE 5:** Results of regression analysis.

Var	Model 1	Model 2	Model 3	Model 4	Model 5	Model 6
	EI	LI	EI	LI	EI	LI
KR			0.454***	0.254**	0.424***	0.246*
TL					0.243	0.382**
KR * TL					–0.371*	–0.436***
GEN	–0.041	–0.022	–0.041	–0.012	–0.009	0.033
JT	0.036	0.135	0.039	0.151	0.028	0.081
JP	–0.009	–0.047	–0.016	–0.056	–0.034	–0.068
ES	–0.088	0.005	–0.054	0.021	–0.042	0.029
Owner	0.133	0.011	0.053	–0.034	0.052	–0.033
R&D	0.267***	0.208**	0.169**	0.172*	0.153**	0.117
*N*	209	209	209	209	209	209

***P < 0.001, **P < 0.01, *P < 0.05.

### Moderating effects

**FIGURE 2:**
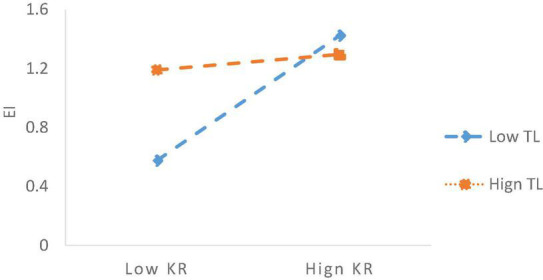
The moderating effects of TL on the link between KR and EI.

In Table 5, Model 5 and Model 6 respectively tested the moderating effects of TL on the relationships between KR and EI, KR and LI. In particular, on the basis of Model 3, Model 5 added the interaction term of KR and TL, and the results of Model 5 shown that the regression coefficient of KR*EI was 0.424 when controlled the Gen, JT, JP, ES, Owner, and R&D. The coefficient of the interaction term of KR and TL on EI is –0.371, *p* < 0.05, suggesting that the contribution of knowledge redundancy (KR) to exploratory innovation (EI) will be diminished under a high level of transactional leadership. Therefore, it is proved that TL negatively moderates the link between KR and EI. Hence, H3 was verified. Moreover, we plotted the slope figure to vividly depict the moderating effect of TL on KR and EI in Figure 2

**FIGURE 3:**
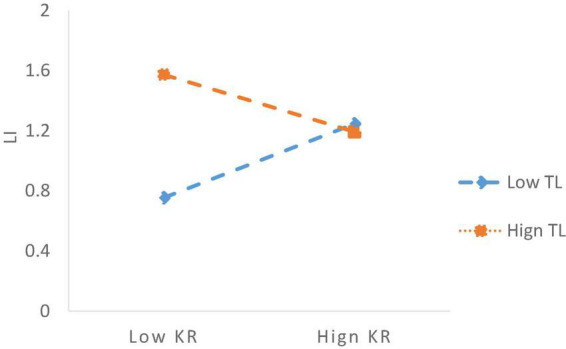
The moderating effects of TL on the link between KR and LI.

Model 6 verified the moderating effect of TL on the relationship between KR and EI. Based on the Model 3, the interaction term of KR and TL was introduced in Model 6. The results suggested that the coefficients of the interaction term of TL*KR on EI was 0.246 when involving the control variables of Gen, JT, JP, ES, Owner, and R&D. Notably, the coefficient of TL*KR on EI was –0.436, *p* < 0.001, suggesting that the contribution of KR to LI will be diminished under a high level of transactional leadership style. Therefore, it is evident that transactional leadership plays a negative moderating role between knowledge redundancy and exploitative innovation. Hence, H4 was verified. Accordingly, we plotted the slope figure to vividly depict the moderating effect of TL on the link between KR and LI in Figure 3.

## Conclusion and discussion

### Conclusion

This study established a theoretical model to shed light on the influence mechanism of knowledge redundancy on innovation ambidexterity and the regulatory effect of transactional leadership on the above relationship. For empirical analysis, we collected data from employees in eastern, central, and western China across varied industries. In line with our proposed hypothesis, we put forward the following major research findings.

First, knowledge redundancy is positively associated with enterprises’ exploratory and exploitative innovation. In challenging and dynamic markets, knowledge redundancy can be used as a reserve resource for enterprises to deal with the external changes, alleviate the resource shortage, and reduce the cost of knowledge acquisition, thereby facilitating exploratory innovation to develop new products and services while attracting new customers. Meanwhile, knowledge redundancy is likely to prompt enterprises to conduct exploitative innovation given that the cost of using existing knowledge to improve existing products or services is far less than the cost of acquiring knowledge from outside sources. To sum up, we posit that a high level of knowledge redundancy effectively promotes enterprises’ exploratory and exploitative innovation and lessens the resource competition between exploratory and exploitative innovation.

Second, transactional leadership weakens the role of knowledge redundancy in promoting exploratory and exploitative innovation. When the leadership style is characterized by transactional, the enterprise’s employees are risk averse, and they would only perform within the range of their job description, because they are afraid of being punished by making faults in work. In this case, employees are less willing to mine existing knowledge or conduct high-risk exploratory activities to innovation. Similarly, if the enterprise’s leader and managers hold a transactional leadership style, they determine their employees’ compensation based on the number of contributions. Given that transactional leadership highlights a reciprocal exchange of benefits between leader and subordinates, employees would be less likely to implement exploitative innovation that yields lower returns.

### Theoretical contributions

On the one hand, this study enriches and expands the knowledge-based theory and ambidexterity theory by providing a better understanding regarding the concept of knowledge redundancy, discussing the influence mechanism of knowledge redundancy in innovation ambidexterity. Based on the findings from previous literature, we further defined knowledge redundancy as the degree of overlap and similarity of an enterprise’s knowledge base, which is acquired through social activities and beyond the current innovation needs. In this sense, this study supplements and improves the previous theoretical frameworks regarding knowledge redundancy, which is conducive to providing new insights on knowledge redundancy theory. In addition, compared with previous knowledge sharing and knowledge transfer, we creatively link knowledge redundancy with innovation ambidexterity (exploratory and exploitative innovation) and explore the relationship between knowledge redundancy and exploratory and exploitative innovation, which is of great significance to the improvement path of innovation from the knowledge management perspective.

On the other hand, this study contributes to the studies of leadership on organizational performance by investigating the moderating effect of transactional leadership on the relationship between knowledge redundancy and innovation ambidexterity. Extant studies have pinpointed the critical role of transactional leadership on enterprises knowledge management and innovation activities from the perspectives of knowledge transfer and knowledge sharing. However, in theoretical research, transactional leadership is usually used as a leading variable, and few studies use it as a regulating variable to study the relationship between knowledge management and enterprise innovation. In light of this, we focused on knowledge redundancy and introduced transactional leadership into the research framework with regard to theories of transactional leadership and innovation ambidexterity, which deepens the studies of leadership while proving its critical role in knowledge management and innovation.

### Managerial implications

Managers should attach special attention to the enterprise’s knowledge redundancy and make full use of the redundant knowledge to promote innovation ambidexterity. First, compared with the previous neglect of knowledge redundancy, managers should pay full attention to the existence of knowledge redundancy, aware the conditions regarding the enterprise’s redundant knowledge, and on this basis, encourage subordinates to use knowledge redundancy to provide knowledge reserves for innovation, and encourage their subordinates to proactively share and exchange knowledge and information. It is noted that a great number of external knowledge search is not a necessary condition for exploratory innovation, because the existing knowledge base, for example, knowledge redundancy is significant to innovation performance. Once it is correctly and sufficiently utilized, knowledge redundancy can provide valuable resources for implementing exploratory and exploitative innovation, alleviating the resource conflict between these two activities, and maintaining long term development. In the innovation processes, managers should encourage their employees to fully mine the existing knowledge base to leverage redundant knowledge to provide knowledge resources for improving organizational performance, such as innovation ambidexterity.

It is important for enterprises to adopt an optimal leadership style, giving employees a certain degree of organizational autonomy to mobilize their intrinsic motivations for innovation. Compared with an open leadership style, the transactional leadership style pays more attention to the stability of daily work affairs and the realization of the organizational goals. In terms of enterprises that managed to pursue high performance of exploratory and exploitative innovation at the same time, a high level of transactional leadership style may not be the ideal one. In addition, we highlight that transactional leadership style pays more attention to the external needs of subordinates rather than encouraging them to conduct extra-role behavior, which is not conducive to stimulating employees’ willingness to design and implement innovation activities. Therefore, if the enterprise current business strategies emphasizing on innovation improvement, managers should adapt to a more open leadership style that enhances the flexibility and transformativeness of organizational regulation and criteria. Moreover, managers should increase direct and effective communications with their subordinates while maintaining a certain dignity, which contributes to a favorable atmosphere that motivate employees for voluntary innovation activities.

### Limitations and future research direction

This study has certain limitations and also provides interesting future research directions. First, the sample size could be expanded for survey data collection and empirical analysis. Considering the operability and controllability, this study collects primary data from employees across various industries in the eastern, central, and western regions of China. As a result, there are inevitably subjective cognitive biases regarding the research sample. Future studies can further expand the scope of research objects, increase the number of research respondents, or focus on a specific sector, providing a more comprehensive understanding of the research topic and improving the validity and scientificity of the proposed questionnaire.

Given this study’s research sample focused on employees in Chinese enterprises, the research conclusion and managerial implications would only be applicable and insightful in emerging economies. In this sense, we advocate future studies to conduct duplicative studies in a different context. For example, it is suggested to set the research setting in enterprises from developing countries with varying types of leadership styles. Moreover, future scholars can also change the research model from other social and psychological factors, such as ambidextrous leadership, psychological capital and psychological contract. The inclusion of new important factors may assist to better understand knowledge management and innovation from the perspective of organizational psychology.

## Data availability statement

The original contributions presented in this study are included in the article/supplementary material, further inquiries can be directed to the corresponding authors.

## Author contributions

YD was responsible for writing—original draft, formal analysis, methodology, and conceptualization of this study. WL contributed to writing—original draft, resources, conceptualization, and proofreading. SW assisted in resources, writing—review and editing, and validation. MY and CM made efforts in writing—review and editing. All authors contributed to the article and approved the submitted version.

## Appendix

**APPENDIX TABLE A1:** The demographic profile of the research sample.

Variable	Category	Frequency	Percent%	Variable	Category	Frequency	Percent%
Gender	Male	110	52.60	Enterprise Scale	20 Employees or Less	38	18.2
	Female	99	47.40		20–100 Employees	64	30.6
Job Position	General Staff	86	41.1		100–300 Employees	38	18.2
	Low-lever Managers	50	23.9		More than 300 Employees	69	33 33
	Middle-level Managers	50	23.9	Ownership	State-owned enterprise	104	49.8
	Senior Managers	23	11.0		private enterprises	66	31.6
Job Tenure	Less than 1 year	29	13.9		Sino-foreign joint venture	19	9.1
	1–3 years	59	28.2		Wholly foreign-owned enterprise	11	5.3
	4–6 years	29	18.7		Other	9	4.3
	7–9 years	39	13.9				
	10 years and over	53	25.4				
